# Cutaneous anthrax outbreak associated with handling dead animals, Rhino Camp sub-county: Arua District, Uganda, January–May 2018

**DOI:** 10.1186/s42522-021-00040-z

**Published:** 2021-04-28

**Authors:** Vivian Ntono, Daniel Eurien, Lilian Bulage, Daniel Kadobera, Julie Harris, Alex Riolexus Ario

**Affiliations:** Uganda Public Health Fellowship Program, Ministry of Health, Kampala, Uganda

## Abstract

**Background:**

On 18 January 2018 a 40 year old man presented with skin lesions at Rhino Camp Health Centre. A skin lesion swab was collected on 20 January 2018 and was confirmed by PCR at Uganda Virus Research Institute on 21 January 2018. Subsequently, about 9 persons were reported to have fallen ill after reporting contact with livestock that died suddenly. On 9 February 2018, Arua District notified Uganda Ministry of Health of a confirmed anthrax outbreak among humans in Rhino Camp sub-county. We investigated to determine the scope and mode of transmission and exposures associated with identified anthrax to guide control and prevention measures.

**Methods:**

We defined a suspected cutaneous anthrax case as onset of skin lesions (e.g., papule, vesicle, or eschar) in a person residing in Rhino Camp sub-county, Arua District from 25 December 2017 to 31 May 2018. A confirmed case was a suspected case with PCR-positivity for *Bacillus anthracis* from a clinical sample. We identified cases by reviewing medical records at Rhino Camp Health Centre. We also conducted additional case searches in the affected community with support from Community Health Workers. In a retrospective cohort study, we interviewed all members of households in which at least one person had contact with the carcasses of or meat from animals suspected to have died of anthrax. We collected and tested hides of implicated animals using an anthrax rapid diagnostic test.

**Results:**

We identified 14 case-patients (1 confirmed, 13 suspected); none died. Only males were affected (affected proportion: 12/10,000). Mean age of case-persons was 33 years (SD: 22). The outbreak lasted for 5 months, from January 2018–May 2018, peaking in February. Skinning (risk ratio = 2.7, 95% CI = 1.1–6.7), dissecting (RR = 3.0, 95% CI = 1.2–7.6), and carrying dead animals (RR = 2.7, 95% CI = 1.1–6.7) were associated with increased risk of illness, as were carrying dissected parts of animals (RR = 2.9, 95% CI 1.3–6.5) and preparing and cooking the meat (RR = 2.3, 95% CI 0.9–5.9). We found evidence of animal remains on pastureland.

**Conclusion:**

Multiple exposures to the hides and meat of animals that died suddenly were associated with this cutaneous anthrax outbreak in Arua District. We recommended public education about safe disposal of carcasses of livestock that die suddenly.

## Introduction

Anthrax is an acute infection caused by *Bacillus anthracis (B. anthracis)*, an aerobic, spore-forming Gram-positive bacteria that can infect both humans and animals [1]. Animals become infected following ingestion of *B. anthracis* spores while grazing in contaminated areas or by eating contaminated feeds. Ingested spores are transformed in vivo into vegetative bacilli that cause disease. When the animal dies, the contaminated carcasses and infectious fluids re-contaminate the environment. The sporulation makes *B*. *anthracis* resistant to degradation in the environment, and spores can persist for extended periods of time, even under adverse conditions [2]. Human cases occur when people are exposed to infected animals. There are three main forms of human anthrax infection, depending on the route of exposure: cutaneous, gastrointestinal, and pulmonary (inhalational) anthrax [3]. The most common, cutaneous anthrax, accounts for approximately 95% of cases [4]. Between one and 12 days after exposure, clinical signs of cutaneous anthrax infection appear as one or more painless, itchy papules or vesicles on the skin, typically on exposed areas such as the face, neck, forearms, or hands. Within 7–10 days of the initial lesion, the papule forms an ulcer, which subsequently crusts over, forming a painless black eschar that is the hallmark of cutaneous anthrax. Localized swelling, painful swollen regional lymph nodes and systemic symptoms may also be present [5]. Without treatment, the case-fatality rate of cutaneous anthrax is 20% [6]; however, it can self-resolve.

Anthrax is endemic in most sub-Saharan African countries [4]. Uganda has been reporting anthrax cases and deaths in humans and animals, including wildlife, since at least 1959 [7, 8]. Anthrax outbreaks in humans have been reported from every region of Uganda, mostly among communities that rear cattle [9]. Surveillance data in Uganda in 2018 revealed 186 reported human cases and 721 reported livestock deaths due to anthrax [9].

On 17 December 2017, a cow suddenly died in Rhino Camp. On 18th January 2018 a 40-year-old man presented with skin lesions at Rhino Camp Health Centre. A skin lesion swab was collected on 20 January 2018 and was confirmed by PCR at Uganda Virus Research Institute on 21 January 2018. Subsequently, at least 9 persons were reported to have fallen ill after reporting contact with livestock that died suddenly. On 9 February 2018, Arua District notified the Uganda Ministry of Health of a confirmed anthrax outbreak among humans in Rhino Camp sub-county. On 11 February, a multi-disciplinary team was sent to investigate and respond to the outbreak. We investigated to determine the source and scope of the outbreak, identify exposures associated with transmission, and recommended evidence-based control and prevention measures.

## Methods

### Study area

Arua District is located in Northwestern Uganda and is bordered by the Democratic Republic of the Congo (DRC) to the west. The district has a total population of about 782,000 persons [10]. The main economic activities in Arua District include cross-border trade with South Sudan and DRC, agriculture, and livestock farming, characterized by significant movement of livestock into and out of the district. Arua District has 18 sub-counties. Rhino Camp sub-county is occupied by both refugees (mostly from DRC) and Ugandan nationals and is named for its proximity to a Ugandan national park which contained white rhinos.

### Case definition and case-finding

We defined a suspected cutaneous anthrax case as onset of skin lesions (e.g., papule, vesicle, or eschar) in a person residing in Rhino Camp sub-county, Arua District from 25 December 2017 to 31 May 2018. We defined a confirmed anthrax case as a suspected case with PCR-positivity for *Bacillus anthracis* from a clinical sample (swab from skin lesions/vesicles, or blood samples).

To identify cases, we reviewed medical records at Rhino Camp Health Centre III. We also conducted additional case searches in the affected community with support from Community Health Workers. We developed a line list of cutaneous anthrax case-persons with patient age, sex, residence, date of onset of signs and symptoms, laboratory investigations, specimens collected, and coordinates of the case-persons’ households.

### Descriptive epidemiology

We performed descriptive epidemiology on the line-listed case-persons. Using an epidemic curve, we described the case-persons by time of onset. Using population data obtained from the district population office, we computed affected proportions (AP) by age-group, sex, and parish. We also drew a choropleth map using QGIS software version to describe case-persons by parish.

### Hypothesis generation

We interviewed 14 suspected case-persons. The key exposures that we explored were those that occurred from 25 December 2017 onwards, including carrying a dead animal to a slaughter site, skinning of a dead animal, dissecting a dead animal, carrying already-dissected parts of dead animals, preparation and cooking of meat of dead animals, contact with hides through skinning and preparation, and having contact with soil through digging.

### Retrospective cohort study

To identify specific animal-related exposures that increased risk for cutaneous anthrax among humans, we formed a cohort among all members of households in which at least one person had contact with the carcass of or products from any animal suspected to have died of anthrax.

### Laboratory investigations

We collected 9 skin lesion swabs from patients with suspected cutaneous anthrax and shipped the samples to the Uganda Virus Research Institute (UVRI; Entebbe, Uganda) for testing. The skin lesion swabs and blood specimens were tested at UVRI using rPCR following a standard protocol developed for nasal swabs [11].

In addition to collecting and testing swabs and blood samples from case-persons, we also tested hides from three implicated cows (hides from cows reported to have died suddenly) using an InBios Active Anthrax Detect™ (AAD) (Anthrax Rapid Test lateral flow immunoassay). The AAD is a point-of-care assay that is under investigational use for detecting *Bacillus anthracis* capsular polypeptide (polyglutamic acid) in suspect animal cases [12]. It was developed as a test for presumptive human inhalation of Anthrax spores [13]. We placed the sample in 600 μL of sterile phosphate buffered saline, vortexed for 10 s, and, after pipetting the solution multiple times, applied 10 μL to the AAD cassette.

Specimens from the dried hides were shipped to the US Centers for Disease Control and Prevention (CDC; Atlanta, GA, USA) for confirmatory testing. DNA extraction from the specimens was performed using a QIAGEN Blood Mini Kit [10]. The resulting DNA was tested using real-time reverse transcription PCR for *B. anthracis* from the Laboratory Reference Network. A formalin-fixed sample from the dried hide was routinely processed, embedded in paraffin, and stained with hematoxylin and eosin, Lillie-Twort Gram stain, and Warthin-Starry silver stain. Immunohistochemistry assays using mouse monoclonal antibodies targeting the *B. anthracis* cell wall and capsule were performed by using an immuno-alkaline phosphatase polymer system as previously described [14, 15].

### Environmental assessment

We observed the possible sites of animal infection, including grazing land and kraals. We mapped out all the kraals in Ombeniya village, identified communal grazing points, and observed both kraals and grazing points for evidence of remains of dead animals. We evaluated the carcass disposal methods on the grazing land. We also looked for indications of human digging activities at points where sudden animal deaths had occurred.

### Data analysis

We used Epi-info Version 7 for data analysis. Descriptive analysis was conducted by person, place, and time, and results were summarized using affected proportions, an epidemic curve, and maps. To measure the associations between exposure variables and illness status, we estimated risk ratios (RR) and their 95% confidence intervals.

### Ethical considerations

Approval to conduct this investigation was sought from the Ministry of Health of Uganda through the office of the Director General Health Services. The Division of Global Health Protection, Centers for Disease Control and Prevention determined that this investigation was not human subjects’ research. Verbal consent was obtained from case-persons and other household members 18 years or older. For participants < 18 years, we sought verbal assent after consent from their parents or guardians. We also sought permission from the local authorities to undertake the outbreak response. Privacy was secured by conducting interviews in a secure place, where none of the people around the home could follow the interview. The questionnaires were kept under lock and key to prevent disclosure of personal information of the respondents to individuals who were not part of the investigation.

## Results

### Descriptive analysis

In total, 14 case-persons were identified by May 2018; none died. One case-person was confirmed by PCR. The mean age of the case-persons was 33 years (SD: 22). Persons aged ≥65 years were the most affected (AP: 27.8/10,000) followed by persons aged 14–64 years (AP: 7.4/10,000), and 5–13 years (AP: 2.8/10,000). The overall affected proportion was 5.8 per 10,000. Only males were affected (AP: 11.9/10,000; Table 1).

**Table 1:** Distribution of cutaneous anthrax case-persons by age and sex in Rhino-camp Sub-County, Arua District, January–May 2018

Characteristic	N	Population	Affected Proportion (/ 10,000)
**Sex**			
Male	14	11,756	12
Female	0	12,235	0
**Age (years)**			
5–13	3	11,036	3
14–64	9	12,235	7
≥ 65	2	720	28

**Fig. 1:**
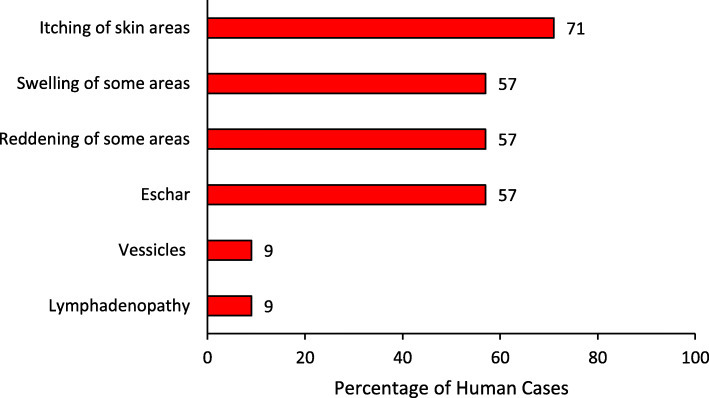
Distribution of cutaneous anthrax signs and symptoms among 14 case-persons in Rhino Camp Sub-county, Arua District, Uganda, January–May, 2018

**Fig. 2:**
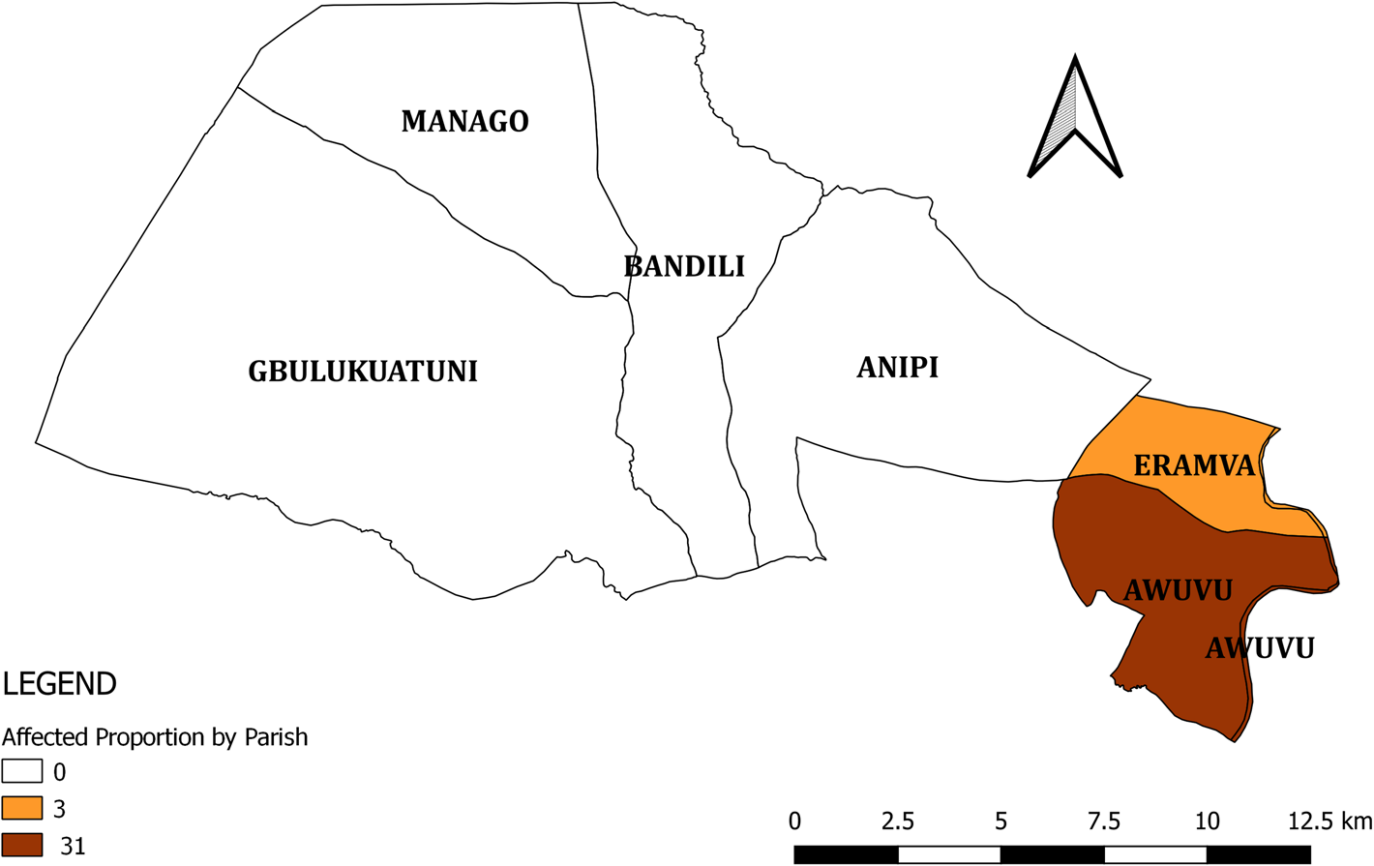
Parish affected proportions (AP) per10, 000 persons for cutaneous anthrax in Rhino Camp, Arua District, Uganda, 2018

Of the 14 case-persons, 10 (71%) presented with itching of skin areas, eight (57%) had swelling or reddening of some areas of the skin, and eight (57%) had eschar formation (Fig. 1). Awuvu Parish was more affected (AP: 31/10,000) compared to Eranva Parish (AP: 2/10,000; Fig. 2). Thirteen cases were from Ombeniva village in Awuvu Parish, while one case was from Eranva Parish, who had come to visit in Ombeniva village on 1 April 2018 and fell ill on 25 April 2018. Therefore, we decided to form the cohort from persons in Ombeniva village.

**Fig. 3:**
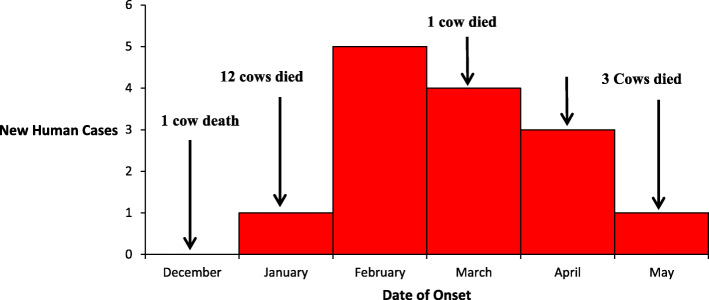
Epidemic curve of case-persons with cutaneous anthrax in Rhino Camp, Arua District, Uganda, 2018

The outbreak lasted for 5 months, from January–May 2018. Cases peaked in February and sharply declined in May (Fig. 3). There was at least one animal death every month except for February. One cow died in December 2017, 12 cows died in January 2018, one cow died in March, one goat died in April, and three cows died in May (Fig. 3).

### Retrospective cohort study findings

The cohort included all members of households in Ombeniva village in which at least one household member had contact with the carcass of or products from an animal suspected to have died of anthrax (*n* = 31). Skinning a dead animal (RR: 2.7, 95%CI 1.1–6.7), dissecting a dead animal (RR: 3.0, 95% CI 1.2–7.6), carrying a dead animal to a site for skinning and dissection (RR: 2.7, 95% CI 1.1–6.7), carrying already-dissected parts of a dead animal (RR: 2.9, 95%CI 1.3–6.5), and preparing and cooking meat from a dead animal (RR = 2.3, 95% CI 0.9–5.9) were all associated with infection (Table 2). All cohort members reported contact with soil.

**Table 2:** Cases of cutaneous anthrax (suspected and confirmed) among household members exposed to carcasses of or products from animals suspected to have died of anthrax in Rhino-camp Sub-County, Arua District, January–May 2018

	Exposed			Not Exposed				
Exposure	Cases	Total	AP (%)	Cases	Total	AP (%)	Risk Ratio	95% CI
Skinning dead animal	10	15	67%	4	16	25%	2.7	1.1–6.7
Dissecting dead animal	10	14	71%	4	17	23%	3.0	1.2–7.6
Carrying dead cow to site for skinning & dissection	10	15	67%	4	16	25%	2.7	1.1–6.7
Carried already dissected parts of dead animal	9	12	67%	5	19	25%	2.9	1.3–6.5
Preparation and cooking of dead meat	10	16	62%	4	15	27%	2.3	0.9–5.9

### Environmental assessment findings

Animal remains were found in the communal grazing land, which indicated possible death or slaughtering of animals within communal grazing areas. Animals were also reported to have died suddenly within the kraals and the communal grazing land. Digging activities were carried out near and within the grazing land; however, this activity was not identified as a risk factor for cutaneous anthrax in this outbreak.

### Laboratory findings

Among nine human skin lesion swabs collected, one (11%) tested positive for *B. anthracis* DNA by PCR at UVRI. The remaining 8 blood samples were negative for *B. anthracis* by PCR at UVRI. It should be noted that, at the time of specimen collection, all patients had already started and some had completed antimicrobial treatment. All 3 samples from the dried hides from the implicated cows tested positive by AAD in the field and were confirmed to be positive for *B. anthracis* by both rPCR and immunohistochemistry at CDC.

## Discussion

Our epidemiological, environmental, and laboratory investigations revealed a cutaneous anthrax outbreak in Arua District, Uganda, associated with handling dead animals. Uganda has reported 14 anthrax outbreaks among humans previously in Western, Eastern, and West Nile regions, where animal husbandry is a major source of income [9]; the most recently preceding outbreak to the one reported here occurred in West Nile Region occurred in Arua in 2017 [9]. All documented anthrax outbreaks in humans in Uganda have occurred within areas with nomadic pastoralism and cattle-rearing (“the cattle corridor”) and have been mainly triggered by physical contact with sick animals through slaughtering, handling, and consumption of dead animals [9]. Since January 2016, an increase in animal movements from other districts within the cattle corridor into Arua District has occurred as ‘Balaalo’ herdsmen have been evicted from their home areas and have brought their animals to graze and drink along the Albert Nile in Arua District [16]. It is believed that these herdsmen have been moving from other areas known to be at high risk for anthrax, such as western Uganda and Karamoja regions where anthrax cases have been reported previously [9]. It is possible that this movement led to an increase in spores in Arua District through influx of infected animals into the area.

This outbreak was associated with a variety of exposures to dead animals or and products from animals suspected to have been infected with anthrax. Such exposures have frequently been associated with cutaneous anthrax, both in Uganda and elsewhere [17–19]. Although at least some in the community are aware of the dangers of handling or consuming animals that die ‘naturally’, poverty in the community may override decisions to forego meat after the financial loss of an animal [20]. A similar anthrax outbreak investigation in India also suggested that persons in poor areas may be hesitant to discard dead animals, even if they have not been slaughtered in a way considered safe [21].

The retrospective cohort analysis estimated that about 4 people with skin lesions were not directly exposed to affected animals or their products. From our findings the disease occurrence is substantively higher in the group directly and knowingly exposed to affected animals and their products compared to the unexposed group; therefore, contact with the carcasses of or products from animals suspected to have died of anthrax was deemed to be high-risk for the development of cutaneous anthrax.

In our investigation, only males were affected. Men are typically the primary persons involved in slaughtering, skinning, and carrying dissected parts of an animal in Uganda, as well as sometimes roasting meat, and have been documented to be more affected than women in similar outbreaks [22]. Children were least affected, likely due to their lack of a role in animal processing or cooking.

Our investigation revealed that there were sudden animal deaths every month except February 2018, both within the kraals and on the grazing land. These deaths coincided with the outbreak among humans. It seems likely that slaughtering of these animals within the grazing land and the kraals, combined with failure to dispose of their carcasses, might have led to contamination of the grazing land and kraals with anthrax spores. This in turn might have facilitated further transmission, as animals subsequently grazed the land during this period. This is a well-known mode of transmission [2, 23].

Our investigation had some limitations. We did not collect soil samples during the environmental assessment to confirm the presence of anthrax spores in the environment. Furthermore, *B. anthracis* was confirmed by rPCR in only 1 out of the 9 skin lesion swab specimens. Blood samples from all 9 patients were also negative for *B. anthracis* by PCR. The reason for this is unknown but is likely related to the fact that all patients had already undergone antibiotic treatment at the time of blood sample collection. In contrast, the only positive skin lesion was from the first sample sent for testing by the district health office to confirm the outbreak and came from a patient who had not yet initiated antibiotic treatment. A point-of-care test for anthrax could facilitate rapid diagnosis in the field; however, such a test is not yet commercially available.

## Conclusions and recommendations

This investigation highlights that cutaneous anthrax is a real risk among persons handling the carcasses and products of animals that die suddenly in Uganda. Our findings support the following recommendations: public education for high-risk communities regarding safe disposal of carcasses of animals that die suddenly; consideration of routine vaccination of healthy animals against anthrax; antibiotic administration to all cutaneous anthrax cases and prophylaxis to exposed persons; and use of rapid diagnostic tests at the district level to quickly provide presumptive evidence of anthrax in animal carcasses and their products to increase safety measures for carcass disposal and to protect at-risk communities.

## Abbreviations

AAD: Active Anthrax Detect
AP: Affected Proportion
CI: Confidence Interval
CHW: Community Health Worker
DRC: Democratic Republic of Congo
RR: Risk Ratio
PCR: Polymerase Chain Reaction
QGIS: Quantum Geographical Information System
SD: Standard Deviation
